# Combining Carbon Nanotubes and Chitosan for the Vectorization of Methotrexate to Lung Cancer Cells

**DOI:** 10.3390/ma12182889

**Published:** 2019-09-06

**Authors:** Giuseppe Cirillo, Orazio Vittorio, David Kunhardt, Emanuele Valli, Florida Voli, Annafranca Farfalla, Manuela Curcio, Umile Gianfranco Spizzirri, Silke Hampel

**Affiliations:** 1 Leibniz Institute of Solid State and Material Research Dresden, 01069 Dresden, Germany (D.K.) (S.H.); 2 Department of Pharmacy, Health and Nutritional Sciences, University of Calabria, 87036 Rende (CS), Italy (A.F.) (M.C.) (U.G.S.); 3 Children’s Cancer Institute, Lowy Cancer Research Centre, UNSW Sydney, NSW 2031, Australia (O.V.) (E.V.) (F.V.); 4 ARC Centre of Excellence for Convergent BioNano Science and Technology, Australian Centre for NanoMedicine, UNSW Sydney, NSW 2052, Australia; 5 School of Women’s and Children’s Health, Faculty of Medicine, UNSW Sydney, NSW 2052, Australia

**Keywords:** multi-walled carbon nanotubes, nanohybrids, methotrexate, pH responsivity, lung cancer

## Abstract

A hybrid system composed of multi-walled carbon nanotubes coated with chitosan was proposed as a pH-responsive carrier for the vectorization of methotrexate to lung cancer. The effective coating of the carbon nanostructure by chitosan, quantified (20% by weight) by thermogravimetric analysis, was assessed by combined scanning and transmission electron microscopy, and X-ray photoelectron spectroscopy (N1*s* signal), respectively. Furthermore, Raman spectroscopy was used to characterize the interaction between polysaccharide and carbon counterparts. Methotrexate was physically loaded onto the nanohybrid and the release profiles showed a pH-responsive behavior with higher and faster release in acidic (pH 5.0) vs. neutral (pH 7.4) environments. Empty nanoparticles were found to be highly biocompatible in either healthy (MRC-5) or cancerous (H1299) cells, with the nanocarrier being effective in reducing the drug toxicity on MRC-5 while enhancing the anticancer activity on H1299.

## 1. Introduction

Lung cancer, one of the most common forms of cancer, is currently the leading cause of cancer-associated death worldwide [1,2]. It can be divided into two types, namely, small-cell lung carcinoma (SCLC) and non-small-cell lung carcinoma (NSCLC) [3,4,5,6], with the asymptomatic early-stage malignancy being responsible for locally advanced or metastatic disease at the time of diagnosis and a poor five-year survival rate (7% and 21% for SCLC and NSCLC, respectively) [7,8].

Although surgery has a major role in the survival of early-stage-cancer patients [9], chemotherapy is still needed to alleviate symptoms and prolongue life [10]. The physiological and pathological features of the organ, together with high variability of the tumor microenvironment, are the main obstacles to the chemotherapeutic treatment of lung carcinomas [11,12], with the insurgence of severe side effects and resistance by cancer cells dramatically limiting the performance of the different therapeutic agents employed in clinics [13].

Consequently, many different delivery methods have been explored as non invasive formulations for a selective and personalized therapy able to optimize the pharmacological efficacy of conventional cytotoxic drugs [14,15,16,17]. Great hope is placed on the role that nanotechnology can help to achieve these results, and different types of nanoparticle systems showed promising results as cancer theranostics [18,19,20].

In particular, carbon nanohybrids, consisting of carbon nanostructures (hexagonal sp^2^-bonded carbon atoms) functionalized with different types of polymers from both synthetic and natural origin, are receiving increasing attention by researchers due to their superior physical, chemical, and biological features [21,22,23,24,25,26,27,28]. Among others, the high specific surface area, the ability to be internalized within cells via passive diffusion and/or endocytosis, and the high drug loading capability due to their rod-like shape, made carbon nanotubes (CNT) an interesting material for the preparation of diagnostic and therapeutic devices [29,30,31,32]. Nevertheless, the great concerns about CNT toxicity, mainly ascribed to their hydrophobic and shape patterns, and resulting in inflammation and alteration of the cellular redox state, generated a debate and reducing their applicability in biomedicine [33,34,35]. The surface functionalization with hydrophilic polymeric materials, increasing CNT water affinity [36,37,38,39,40], was proposed as a valuable approach to reduce its toxicity occurring at different levels within the human body, including the asbestosis-like toxicity to lung fibroblasts [41,42,43,44,45,46].

Among the different polymeric materials proposed for physical CNT functionalization, including proteins and polysaccharides, chitosan (CS) showed peculiar advantages because it coupled the advantages of high biocompatibility, chemical versatility, and tumor tissue affinity, with the easily processability in nanoparticle delivery systems suitable for administration via various routes, especially pulmonary [47,48,49]. CS possessed hydrophilic and cationic behaviors, being suitable for the surface functionalization of carboxylated CNT with the formation of nanocarriers stable in water solution [50,51]. Furthermore, due to its insolubility at pH 7.4 (physiological condition) and solubility in media with pH < 6.5 (tumor microenvironment), a pH-responsive behavior can be achieved [52,53,54], with the carrier able to retain the payload until reaching the target site with negligible early release.

Herein, we exploit the high lung affinity of CNT as a Trojan horse approach for delivering methotrexate (MTX) to the NSCLC. In detail, multi-walled carbon nanotubes (MWCNT) were functionalized with CS (CS_MWCNT), turning the weakness of CNT lung toxicity into the strength of a site-specific carrier with reduced side effects. The effectiveness of the proposed nanohybrids was assessed by extensive characterization of physico-chemical properties, and biological assays using NSCLC H1299 and MRC-5 cells, as models for NSCLC and healthy cells, respectively.

## 2. Materials and Methods

### 2.1. Preparation of CS_MWCNT

MWCNT were synthesized by fixed-bed chemical vapor deposition method, as reported in the literature [55].

For the oxidation procedure, 200 mg CNT were ultrasonicated in a mixture of HNO_3_ (65%) and H_2_SO_4_ (98%) in the volume ratio 1:3 for 3 h [56]. Thereafter, the oxidized MWCNT (ox-MWCNT) were separated by filtration, washed with deionized water until solution neutrality, and finally dried at 108 °C overnight [57].

For the fabrication of CS_MWCNT nanohybrid, 10 mg ox-MWCNT in 5 mL acetic acid (5%) was treated for 30 s with a sonication tip. Then, 25 mg CS (wt 50,000–190,000 Da) was added and the mixture was stirred overnight at room temperature. Thereafter, it was filtrated and washed with 5% acetic acid and then with deionized water until solution neutrality. Finally, nanohybrid was dried at 40 °C overnight.

All chemicals were from Merck/Sigma Aldrich, Darmstadt, Germany.

### 2.2. Characterization Procedure

Scanning electron microscope images were acquired by a NOVA NanoSEM 200 [0–30 kV] (Thermo Fisher Scientific, Hillsboro, OR, USA) by depositing grounded samples onto self-adhesive, conducting carbon tape (Plano GmbH, Wetzlar, Germany).

Transmission electron microscope images were acquired on HRTEM/Tecnai F30 [300 kV] (Thermo Fisher Scientific, Hillsboro, OR, USA) by pressing samples between two small slides of aluminum foil on a Cu TEM grid (200 mesh, Plano GmbH, Wetzlar, Germany).

X-ray photoelectron spectroscopy spectra (0.8 eV resolution) of samples prepared on an aluminum foil were performed using Al Kα radiation on a XPS/PHI 5600-CI (Physical Electronics, Chanhassen, MN, USA).

Raman spectra (2 cm^−1^ resolution) were recorded on a Raman–Fourier transform spectrometer IFS 100 (Bruker Corporation, Ettlingen, Germany), operating at a wavelength of 633 nm with a laser power of 8 mW. Samples were prepared by deposition on an aluminum foil.

Thermogravimetric analysis (~5.0 mg samples) was performed in a Perkin-Elmer TGA-7 analyzer (Perkin-Elmer, Rodgau, Germany) under nitrogen atmosphere (flow of 100 mL min^−1^) and the following heating conditions: from 50 to 100 °C at 10 °C min^−1^; b) 30 min isothermal 100 °C; from 100 to 275 °C at 10 °C min^−1^; 30 min isothermal 275 °C; from 275 to 800 °C at 10 °C min^−1^.

### 2.3. In Vitro MTX Release

1.0 mg MTX (Merck/Sigma Aldrich, Darmstadt, Germany) was loaded on 10 mg CS_MWCNT in 10.0 mL distilled water by stirring at room temperature for 24 h and recovering the loaded sample (MTX@CS_MWCNT) after drying under vacuum.

The MTX release profiles were recorded by using, in separate experiments, release media consisting of phosphate buffered saline (0.01 mol L^−1^, pH 7.4) or acetate buffer (0.1 mol L^−1^, pH 5.0). In a standard procedure, MTX@CS_MWCNT (15 mg) was dispersed into 1.5 mL release media at the selected pH in a dialysis bag (MWCO: 12,000–14,000 Da, VWR International GmbH, Darmstadt, Germany), and dialyzed against 13.5 mL of the corresponding buffer. At predetermined time intervals, the amount of MTX in the releasing media was determined by HPLC analysis.

The HPLC system consisted of a Jasco BIP-I pump operating at a flow rate of 1.0 mL min^−1^ and Jasco UVDEC-100-V detector set at 306 nm. A 250 × 4 mm C-18 Hibar^®^ column, 10 µm particle size (Merck/Sigma Aldrich, Darmstadt, Germany) was used as stationary phase, while the mobile phase consisted of methanol: 0.05% w/w H_3_PO_4_ (Merck/Sigma Aldrich, Darmstadt, Germany) aqueous solution (23/77, vol/vol).

### 2.4. Cytotoxicity Tests

Cell culture conditions were as follows. Roswell Park Memorial Institute (RPMI) medium supplemented with 10% FBS and 1% L-glutamate for human lung cancer cells H1299 (ATCC, In Vitro Technologies Pty. Ltd., Melbourne, Victoria, Australia); Eagle’s Minimum Essential Medium supplemented with 10% FBS for human lung fibroblasts cells MRC-5 (ATCC, In Vitro Technologies Pty. Ltd., Melbourne, Victoria, Australia). Both cell lines were grown as a monolayer in a humidified atmosphere at 37 °C and in 5% CO_2_.

Alamar Blue assay was used to assess effect of 48 h CS_MWCNT, MTX, and MTX@CS_MWCNT treatments. In a typical procedure, cells were plated in clear transparent 96-well plates 24 h prior to treatment, with the optimized cell density being 1.5 × 10^3^ and 5 × 10^3^ cells/well for H1299 and MRC-5, respectively. In all experiments, MTX concentrations (either in the free and in the loaded form) of 7.72 × 10^−5^ and 1.51 × 10^−3^ mg mL^−1^ were used, corresponding to CS_MWCNT concentrations of 7.72 × 10^−4^ and 1.51 × 10^−2^ mg mL^−1^, respectively.

All chemicals were purchased from Merck/Sigma Aldrich, Darmstadt, Germany.

### 2.5. Cell Internalization Studies

To prepare the samples for TEM analysis, human BCa cell line EJ28 (University of Frankfurt, Frankfurt, Germany) were seeded and incubated with 1.51 × 10^−2^ mg mL^−1^ CS_MWCNT for 24 h in Eagle’s Minimum Essential Medium. After co-incubation, the medium was removed and the cells were detached and fixed with 2% glutaraldehyde solution at 4 °C. After washing, cells were dehydrated with increasing concentrations of acetone and embedded in a solution (50:50) of EPOXI resin. Then EPOXI resin was cut in 70 nm thin slices.

The samples were analyzed by transmission electron microscopy (TEM) using a FEI Tecnai T20 microscope (Thermo Fisher Scientific, Hillsboro, OR, USA) and operating at 200 keV.

### 2.6. Statistical Analysis

Experiments were carried out in triplicate. Values were expressed as means ± standard error of the mean. For viability assay, statistical significance was assessed by two-way analysis of variance followed by post-hoc comparison test (Tukey’s test). Significance was set at *p* < 0.01.

## 3. Results and Discussion

### 3.1. Material Properties

The biological performance of a CNT nanohybrid strictly depends on its physic-chemical properties, which are in turn affected by either the organic or inorganic counterpart, opening a broad scenario for the development of engineered materials [38,58]. Thus, the selection of carbon nanostructures with appropriate behaviors is the main challenge to be addressed when designing high performing carrier systems [59,60].

**Figure 1:**
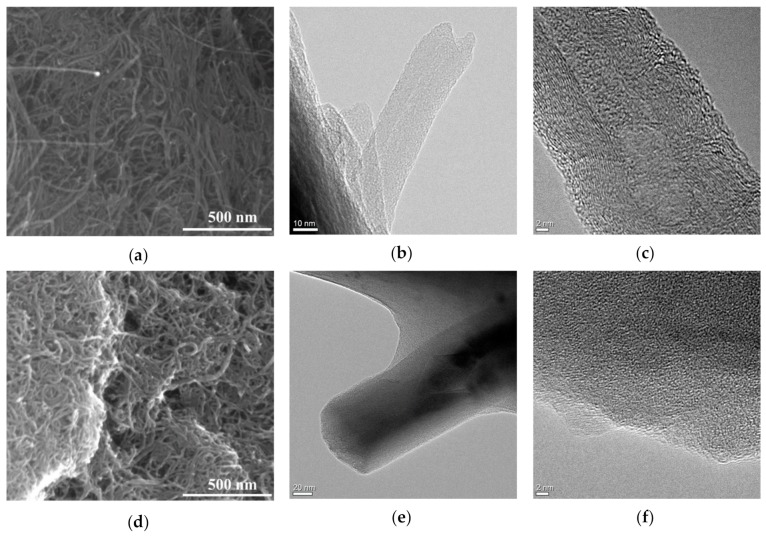
Representative SEM images of (**a**) multi-walled carbon nanotubes (MWCNT); (**d**) chitosan multi-walled carbon nanotubes (CS_MWCNT) and representative high-resolution transmission electron microscopic images of (**b**,**c**) MWCNT and (**e**,**f**) CS_MWCNT.

It is well know that the synthetic and purification procedures play key roles in determining the CNT features, with the possibility to modulate the morphological patterns (e.g., size and number of shells), the electric and magnetic responses, the number of defects and density of branches, and the surface chemical affinity [61,62]. Here, we employed a fixed-bed chemical vapor deposition method, allowing us to produce well defined MWCNT with a narrower size distribution (length in a range of 110–980 nm, average inner diameter of 0.7–1.5 nm, and outer diameter of 5–8 nm corresponding to 4–7 graphene shells; see Figure 1a [63]) compared to MWCNT prepared by different techniques, such as aerosol assisted chemical vapor deposition (length in the range 10–30 µm and average outer diameter of 5–25 nm, corresponding to 20–30 graphene walls [64]). The significantly lower dimensional range allowed a more effective interaction with cell environment of lung, key requirement for an effective drug carrier [65].

The synthetic protocol involved the preliminary MWCNT oxidation by means of H_2_SO_4_/HNO_3_ mixture in order to remove the residual catalyst and form COOH groups suitable for further functionalization.

We proposed a non-covalent coating of ox-MWCNT surface with a polysaccharide material such as CS, which is able to interact with the ox-MWCNT surface via either hydrophobic or hydrophilic interactions. In the first case, the C-C skeleton of sugar repeating units is connected with the sp^2^ carbon layer of CNT, while the amino and hydroxyl functionalities of CS are involved in the formation of electrostatic interactions and hydrogen bonds with the COOH of ox-MWCNT [66,67].

The effectiveness of each step was assessed by a multi-technique approach, involving morphological (combined SEM/TEM analysis) characterization, chemical analysis via X-ray photoelectron spectroscopy (XPS), investigation of vibrational characteristics and structural properties (Raman spectroscopy), and determination of thermal (Thermogravimetric analysis—TGA) features.

Pristine MWCNT show the typical structure with the presence of a few defects due to oxidation (Figure 1a–c), and the polymeric material around them being evident in the observation of the local structure of CS_MWCNT nanohybrid (Figure 1d–f).

**Figure 2:**
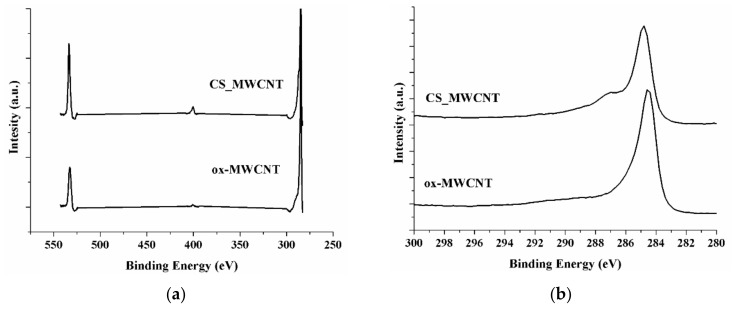
(**a**) XPS survey spectrum of ox-MWCNT, and CS_MWCNT; (**b**) XPS C 1*s* binding energy curves of ox-MWCNT, and CS_MWCNT.

Chemical analysis of samples was accomplished by XPS (Figure 2a). Pristine MWCNT shows only the carbon (C1*s*) signal (data not shown), while in CS_MWCNT hybrid, the oxygen (O1*s*) and nitrogen (N1*s*) signals are also recorded, giving another proof of the formation of nanohybrid. Furthermore, the C1*s* spectrum (Figure 2b) shows two main peaks, located at 284.5 and 285–290 eV, respectively. The first peak corresponds to the C-C and C-H components of either nanotubes and CS backbone [68,69], while the second broad band is assigned to C bonds (e.g., C-O, C-N) within the CS structure [69].

**Figure 3:**
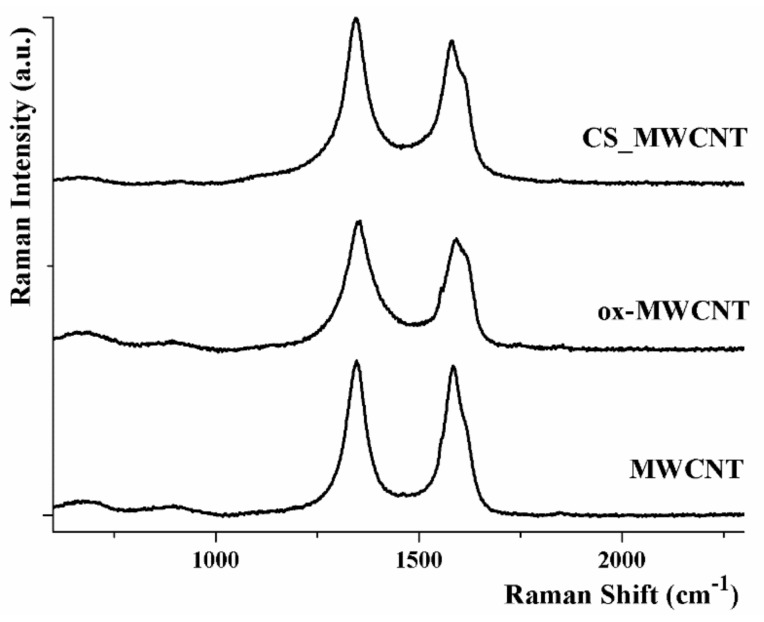
Raman spectra of MWCNT, ox-MWCNT, and CS_MWCNT. For all samples, D and G bands are located at 1347 and 1582 cm^−1^, respectively.

The CS coating was also confirmed by Raman spectroscopy, with the Raman spectra of MWCNT showing the typical signals at 1346 and 1584 cm^−1^, corresponding to the D and G bands, respectively (Figure 3).

D bands originated from disorder and distortions of the carbon network, while G (graphitic) bands corresponded to the tangential (E_2*g*_) stretching mode within the carbon sp^2^ in the CNT rolled graphene sheet [64].

The Raman patterns of ox-MWCNT and CS_MWCNT showed a similar behavior, although D and G bands being in different relative ratios: the I_G_/I_D_ ratio of 0.96 recorded for pristine MWCNT became 0.86 for ox-MWCNT as a consequence of the increased defect sites (mainly COOH groups) upon oxidation. No further change in the I_G_/I_D_ ratio was recorded after CS coating, since the non covalent coating did not involve formation of new defects on ox-MWCNT surface.

**Figure 4:**
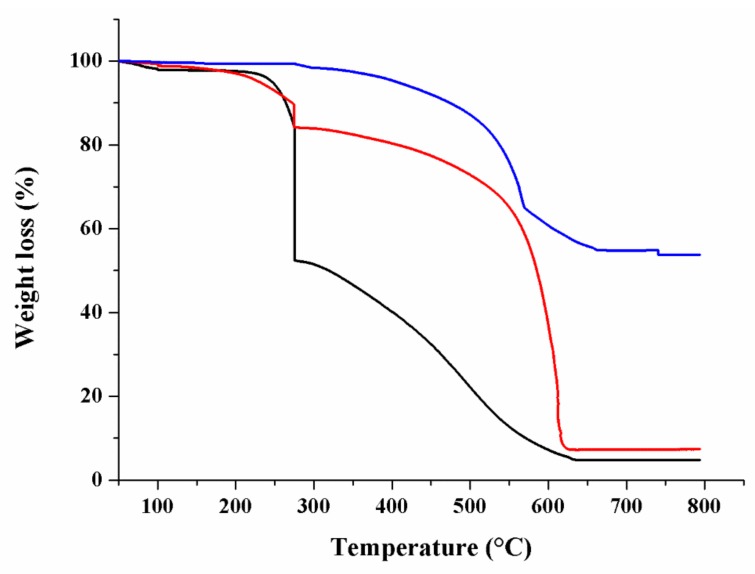
TGA thermograms of ox-MWCNT (blue line), CS (black line), and CS_MWCNT (red line) showing the effect of polymer coating on the thermal stability of hybrid samples.

The presence of CS coating was also evident by TGA measurements, because of the different thermal behavior of CNT and polymer counterparts (Figure 4).

In detail, the weight loss for pristine MWCNT was lower than 5% at 800 °C (data not shown), indicating the absence of relevant defect sites in the graphene layers. The oxidation process by means of acidic treatment (HNO_3_/H_2_SO_4_ mixture) resulted in the creation of more defects sites, with a total weight loss of around 45% at 800 °C (CO_2_ loss) [70]. Pure CS showed two steps thermal degradation, corresponding to the amine side or N-acetyl side groups (275–290 °C) and to the oxidative removal of the glycosidic linkage (600–800 °C), respectively. The TGA curve of CS_MWCNT was similar to that of pure CS, with obvious lower weight loss due to the enhanced thermal stability due to MWCNT. From the difference in the weight losses, the amount of CS coating was calculated and found to be 20% [71].

### 3.2. Drug Release Studies and In Vitro Anticancer Activity

**Figure 5:**
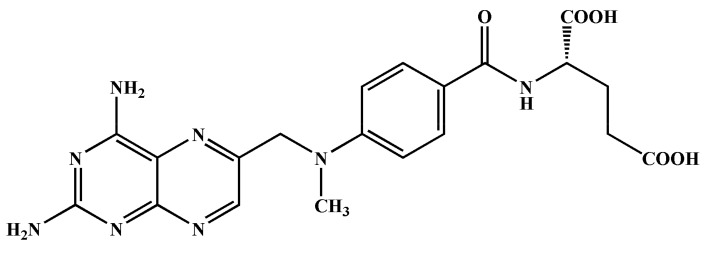
Chemical structure of methotrexate (MTX).

For the treatment of lung carcinomas, MTX (Figure 5) was found to possess high activity due to the strong inhibition of dihydrofolate reductase and folate receptor binding, but its clinical applicability is hindered by the development of multi-drug resistance, as well as from the insurgence of severe side effects to normal tissues and cells [72,73,74].

To date, many nanoparticle systems have been proposed for the vectorization of MTX with the aim to maximize the treatment effectiveness [75,76]. Here, the nanohybrid CS_MWCNT was designed as MTX delivery vehicle which, combining the pH-responsivity of CS with the ability of MWCNT to be internalized rapidly within cells, was expected to vectorize MTX to cancer cells, thus reducing the side effects to normal cells.

**Figure 6:**
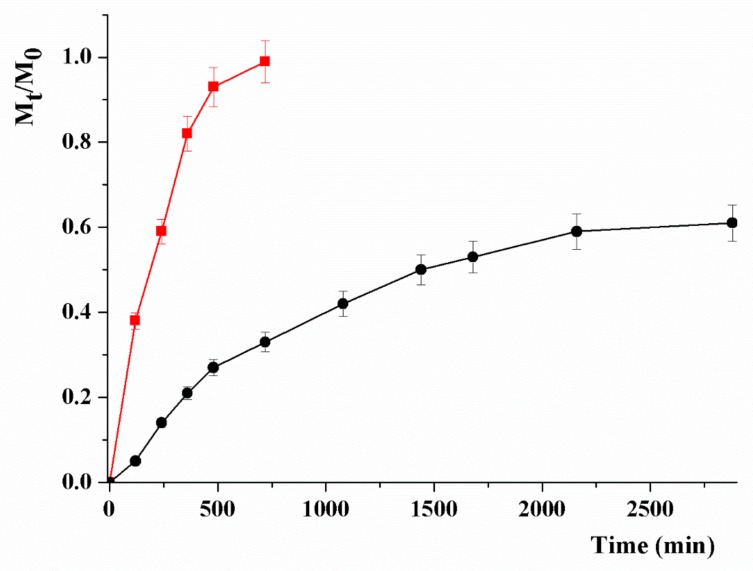
pH-responsive MTX release profile (*M_t_*/*M*_0_) from CS_MWCNT at pH 5.0 (red line) and 7.4 (black line).

MTX was loaded by employing a drug to carrier ratio (by weight) of 10%, and the release profiles recorded at pH 5.5 and 7.4, simulating the endosomal pH of cancer cells, and the normal physiological pH, respectively (Figure 6).

Mathematical modelling of the release profiles allowed us to better highlight the effect of pH on the physicochemical affinity of MTX between the carrier and the release media [77]. This phenomenon can be expressed by the *α* value calculated as follows (Equation (1)):

(1) $$\alpha =\frac{F_{max}}{1-F_{max}}$$

where *F_max_* represents the maximum value of relative release (*M_t_*/*M*_0_).

By the employed kinetic model, the release profiles can be described according to the following Equations (2) and (3):

(2) $$\frac{M_{t}}{M_{0}}=F_{max}\left(1-e^{-\left(\frac{k_{R}}{F_{max}}\right)t}\right)$$

(3) $$\frac{M_{t}}{M_{0}}=\frac{F_{max}\left(e^{2\left(\frac{k_{R}}{\alpha }\right)t}-1\right)}{1-2F_{max}+e^{2\left(\frac{k_{R}}{\alpha }\right)t}}$$

with *k_R_* being the release rate constant.

When experimental data fit with Equation (2), reversible first-order kinetics can be evoked, while reversible second-order kinetics occur when Equation (3) is satisfied.

In our conditions, higher *R*^2^ values were obtained when Equation (2) is applied, suggesting release profiles with predominant first order kinetics (Table 1).

A highly remarkable pH responsivity was recorded, with a higher amount of MTX release at acidic (*F_max_* ~ 1.0) vs. physiological (*F_max_* = 0.65) pH. Further information can be obtained by comparing the kinetic constants and the time required for reaching 50% of *F_max_* ($t_{1/2}$ value, Equation (4)) in the two pH conditions:

(4) $$t_{1/2}=\frac{F_{max}}{k_{R}}ln2$$

This comparison better highlighted the pH responsivity, with the significant enhancement of the releasing rate at pH 5.0 resulting in a four-fold reduction of the *t*_1/2_ value, allowing a tumor specific delivery of the cytotoxic agent to be hypothesized.

**Figure 7:**
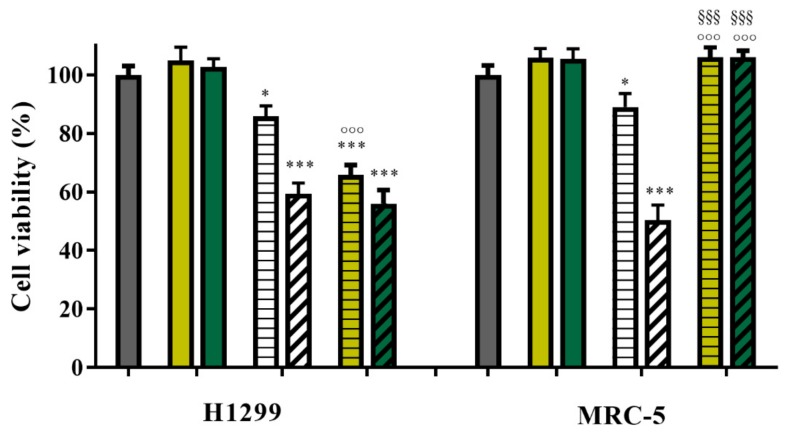
Cell viability of H1299 and MRC-5 cells after treatment with empty CS_MWCNT (filled bar), free MTX (empty streaked bar), and MTX@CS_MWCNT (filled streaked bar). MTX concentrations were 7.72 × 10^−5^ (rowed bar) and 1.51 × 10^−3^ (diagonal bar) mg mL^−1^. CS_MWCNT concentrations were 7.72 × 10^−4^ (light green bar) and 1.51 × 10^−2^ (dark green bar) mg mL^−1^. * *p* < 0.01, *** *p* < 0.0001, vs. corresponding control; °°° *p* < 0.0001 vs. free MTX at equivalent concentration; §§§ *p* < 0.0001 vs. same treatment on H1299 cells.

This finding matched well with the results of cell viability studies performed selecting H1299 cells, NSCLC derived from the lymph node, and MRC-5 cells, fibroblast derived from normal lung tissue, as models for cancer and physiological environments, respectively (Figure 7).

In all experiments, MTX concentrations of 7.72 × 10^−5^ and 1.51 × 10^−3^ mg mL^−1^ and a fixed drug to carrier ratio of 10% (by weight) were employed, thus allowing us to calculate CS_MWCNT concentrations of 7.72 × 10^−4^ and 1.51 × 10^−2^ mg mL^−1^, respectively.

Empty CS_MWCNT was found to do not affect the viability of both cell lines, proving the key requirement of high biocompatibility of any carrier device. Free drug showed the expected concentration-dependent cytotoxicity, with the highest tested concentration resulting in a cell viability reduced to 59% and 50% in H1299 and MRC-5 cases, respectively. The similar effect of the drug on both cells lines is related to the specific metabolic features and high sensibility to almost any types of chemical species of MRC-5 cells, which, indeed, have been recognized as a valid model for the classification and risk assessment studies of chemicals [78,79,80].

Interestingly, the loaded MTX@CS_MWCNT were found to be highly selective in killing cancer cells, with viability of health MRC-5 being not significantly (*p* > 0.01) affected by the treatments at the tested concentrations. In addition, MTX@CS_MWCNT were found to possess equal or even more activity than the free drug on cancer cells. At 7.72 mg mL^−1^, indeed, the H1299 viability was reduced by 15% when free MTX was used as treatment, while the loaded drug significantly (*p* < 0.0001) increased the amount of death cells up to 44%. These results, related to the different metabolic rate of cancer and healthy lung cells, as well as to the different pH values of the two environment differently affecting the MTX release, clearly proved the in vitro efficiency of the CS_MWCNT nanohybrid.

**Figure 8:**
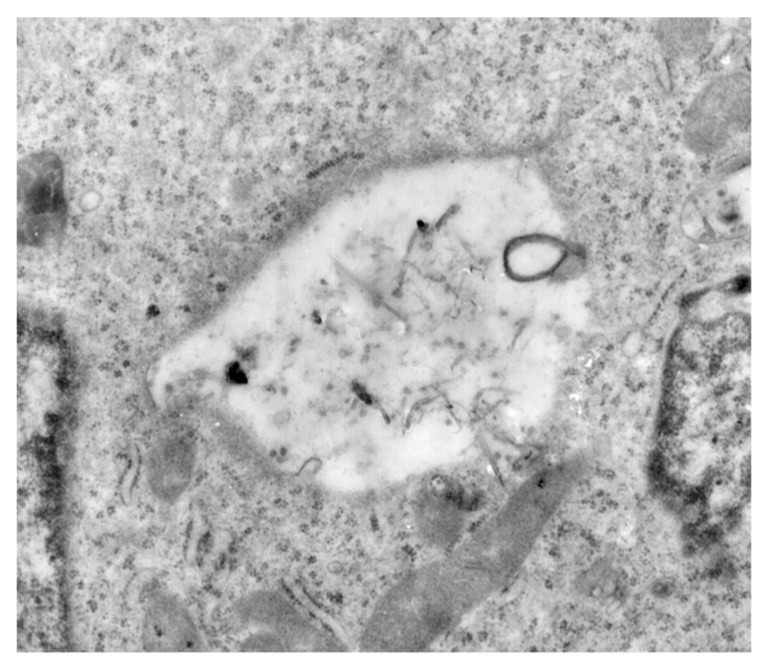
TEM images of EJ28 BCa cells after incubation with 1.51 × 10^−2^ mg mL^−1^ CS_MWCNT, proving the presence of nanohybrid within the cytoplasm.

The findings were also found to be in accordance with data in the literature showing that a slow release of cytotoxic drugs from nanocarrier systems is associated with a reduction of toxicity of loaded vs. free drug at equivalent concentrations [81,82]. Indeed, free MTX penetrated the cell membrane through passive diffusion, while a comparatively longer time was required for endocytosis-mediated internalization of MTX@CS_MWCNT [83,84,85,86]. As a proof of concept of this statement, and with the aim to extend the applicability of the proposed nanocarrier to different cancer cell lines, we investigated and proved the ability of CS_MWCNT to cross the cell membrane and be localized within the cytoplasm of EJ28 BCa cells (Figure 8).

## 4. Conclusions

A novel CS_MWCNT nanohybrid was found to be suitable as a nanocarrier for the selective delivery of MTX to H1299 lung cancer cells, with negligible toxicity to healthy MRC-5 cells. The performance was related to the specific features of the nanohybrid, which possessed high biocompatibility and affinity for the drug, thus determining a sustained and pH-responsive release profile. The extensive physic and chemical characterization of CS_MWCNT allowed us to highlight the correct assembly of organic and inorganic counterparts and the pH responsivity patterns. The in vitro biological characterization showed that, by taking advantage of the peculiar characteristics of lung cells, the nanoparticle carrier was able to synergize the drug activity in cancer cells, while reducing the toxicity of healthy ones.

The results of this study can be considered of great importance when hypothesizing the potential bench to clinic application of the proposed device, although further experiments should better elucidate the therapeutic performance in either different cancer cell lines or appropriate in vivo models, with the determination of the cross-applicability, the pharmacokinetics and toxicity profiles, as well as the ability to reduce tumor volume and prevent metastases.

## Figures and Tables

**Table 1:** *R*^2^ values and kinetic parameters for mathematical modelling of MTX release.

Mathematical Model	Parameter	MTX	
		pH 5.0	pH 7.4
$\frac{M_{t}}{M_{0}}=F_{max}\left(1-e^{-\left(\frac{k_{R}}{F_{max}}\right)t}\right)$	*R* ^2^	0.9906	0.9964
	*k_R_* (10^−3^)	4.31	0.66
	*F_max_*	0.99	0.65
	*α*	99	1.86
	*t*_1/2_	159	681
$\frac{M_{t}}{M_{0}}=\frac{F_{max}\left(e^{2\left(\frac{k_{R}}{\alpha }\right)t}-1\right)}{1-2F_{max}+e^{2\left(\frac{k_{R}}{\alpha }\right)t}}$	*R* ^2^	0.9149	0.6959
	*k_R_* (10^−3^)	11.12	0.72
	*F_max_*	0.99	0.70
	*α*	99	0.33
	*t*_1/2_	88	760
